# Ablation of specific long PDE4D isoforms increases neurite elongation and conveys protection against amyloid-β pathology

**DOI:** 10.1007/s00018-023-04804-w

**Published:** 2023-06-12

**Authors:** Dean Paes, Melissa Schepers, Emily Willems, Ben Rombaut, Assia Tiane, Yevgeniya Solomina, Amy Tibbo, Connor Blair, Elka Kyurkchieva, George S. Baillie, Roberta Ricciarelli, Chiara Brullo, Ernesto Fedele, Olga Bruno, Daniel van den Hove, Tim Vanmierlo, Jos Prickaerts

**Affiliations:** 1 Department of Psychiatry & Neuropsychology, School for Mental Health and Neuroscience, Maastricht University, Maastricht, The Netherlands; 2 Department of Neuroscience, Biomedical Research Institute, Hasselt University, Diepenbeek, Belgium; 3 Institute of Cardiovascular and Medical Sciences, College of Medical, Veterinary and Life Sciences, University of Glasgow, Glasgow, UK; 4 Section of General Pathology, Department of Experimental Medicine, School of Medical and Pharmaceutical Sciences, University of Genoa, Genoa, Italy; 5 IRCCS Ospedale Policlinico San Martino, Genoa, Italy; 6 Section of Medicinal Chemistry, Department of Pharmacy, School of Medical and Pharmaceutical Sciences, University of Genoa, Genoa, Italy; 7 Section of Pharmacology and Toxicology, Department of Pharmacy, School of Medical and Pharmaceutical Sciences, University of Genoa, Genoa, Italy; 8 Department of Psychiatry, Psychosomatics and Psychotherapy, University of Würzburg, Würzburg, Germany

**Keywords:** Alzheimer’s disease, CRISPR/Cas9, Phosphodiesterase 4D, APP/PS1

## Abstract

Inhibition of phosphodiesterase 4D (PDE4D) enzymes has been investigated as therapeutic strategy to treat memory problems in Alzheimer’s disease (AD). Although PDE4D inhibitors are effective in enhancing memory processes in rodents and humans, severe side effects may hamper their clinical use. PDE4D enzymes comprise different isoforms, which, when targeted specifically, can increase treatment efficacy and safety. The function of PDE4D isoforms in AD and in molecular memory processes per se has remained unresolved. Here, we report the upregulation of specific PDE4D isoforms in transgenic AD mice and hippocampal neurons exposed to amyloid-β. Furthermore, by means of pharmacological inhibition and CRISPR-Cas9 knockdown, we show that the long-form PDE4D3, -D5, -D7, and -D9 isoforms regulate neuronal plasticity and convey resilience against amyloid-β in vitro. These results indicate that isoform-specific, next to non-selective, PDE4D inhibition is efficient in promoting neuroplasticity in an AD context. Therapeutic effects of non-selective PDE4D inhibitors are likely achieved through actions on long isoforms. Future research should identify which long PDE4D isoforms should be specifically targeted in vivo to both improve treatment efficacy and reduce side effects.

**Supplementary Information:**

The online version contains supplementary material available at 10.1007/s00018-023-04804-w.

## Background

Memory consolidation deficits associated with Alzheimer’s disease (AD) cause severe impairment in AD patients, and place a tremendous burden on their caretakers and family members. Hence, pharmacological interventions that stimulate the molecular machinery underlying memory consolidation would improve the quality of life of AD patients and people close to them. Cyclic adenosine monophosphate (cAMP) plays a pivotal role as second messenger in signaling cascades that regulate memory consolidation [1]. Enzymatic degradation of cAMP predominantly occurs by phosphodiesterase 4 (PDE4) enzymes [2]. Consequently, inhibition of PDE4 would promote cAMP signaling and associated memory consolidation processes [3]. Accordingly, PDE4 inhibition has been found to enhance memory functioning in both rodents and healthy humans [4–6]. In addition, PDE4 inhibition has shown pro-cognitive effects in AD animal models [7–12].

Despite the therapeutic potential of PDE4 inhibitors to restore memory functioning in AD, clinical use is hindered due to severe side effects, such as nausea and emesis [13]. Interestingly, the PDE4 enzyme family is encoded by four genes (*PDE4A-D*), each producing a sub-family comprising of several isoforms (e.g., PDE4D1-9). By specific inhibition of PDE4 sub-families or isoforms rather than non-selective PDE4 inhibition, adverse effects may be minimized or prevented [3, 13, 14]. The PDE4D subtype has shown to be a promising target for memory enhancement. In a recent exploratory study, PDE4D expression in blood was found to negatively correlate with cognitive function in monozygotic twins [15]. Moreover, *PDE4D* gene variants affect functional brain networks in patients with mild cognitive impairment and AD [16]. In rodents, inhibition of PDE4D, rather than PDE4B, was found to enhance memory functioning [17]. Moreover, in AD mouse models, pharmacological inhibition or shRNA-mediated knockdown of PDE4D has been reported to restore amyloid-β (Aβ) induced memory deficits [18–21].

Recently, the PDE4D-selective inhibitor GEBR32a was designed as a fluorinated derivative of the lead compound GEBR8a [22, 23]. Compared to its parent compound GEBR8a, GEBR32a is very selective for PDE4D isoforms with improved potency, as GEBR32a showed an IC_50_ of 2.43 μM toward PDE4D3, whereas GEBR8a showed an IC_50_ of 7.60 μM [22, 23]**.** Moreover, GEBR32a is characterized by a good toxicological and pharmacokinetic profile, with reduced side effects according to the xylazine/ketamine-induced anesthesia test in mice [22, 24]. We hypothesize this is a consequence of PDE4D isoform selectivity of the GEBR32a compound. Hence, even more specific targeting, i.e., targeting individual PDE4D isoforms, may be needed for an efficacious and clinically safe treatment strategy. Previously, we reported that PDE4D expression is isoform-specifically altered in post-mortem brains of AD patients and that this expression correlates with the degree of pathology and cognitive decline [25]. Specific PDE4D isoforms may therefore contribute more to AD-associated memory deficits and thus be more promising pharmacological targets. Strikingly, the different isoforms encoded by the *PDE4D* gene localize to different intracellular compartments and regulate distinct cAMP signaling domains [2, 13, 26, 27]. Neuronal plasticity, which is modulated by cAMP and involves dynamic neurite growth, is crucial for proper memory consolidation [28, 29]. In AD, neuronal plasticity is found to be heavily impaired [30]. Non-selective PDE4 inhibition promotes neuronal plasticity and can restore Aβ-induced plasticity impairments [31]. Moreover, genetic ablation of PDE4D appears to specifically increase the number of neurites in vivo [32]. These findings indicate that PDE4D regulates cAMP signaling domains involved in neuronal plasticity. Therefore, it is crucial to understand which specific PDE4D isoforms control these processes to identify safe pharmacological targets for memory enhancement in AD.

Here, we investigated how PDE4D isoform expression levels and downstream cAMP-PKA-CREB signaling are affected in transgenic AD mouse brains and in Aβ-exposed mouse hippocampal neurons. Furthermore, by means of pharmacological inhibition and CRISPR-Cas9-mediated knockdown, we determined which individual PDE4D isoforms regulate neuronal plasticity in vitro and whether ablation of specific isoforms can convey resilience against Aβ toxicity.

## Methods

### Animals and behavioral testing

All experimental procedures were approved by the local ethical committee of Hasselt University for animal experiments and met governmental guidelines. Eighteen female wild-type (C57bl/6 OlaHsd) and twenty-two female transgenic Alzheimer mice (APPswe/PS1dE9) were used. Mice were genotyped by PCR analysis of ear biopsies. At the age of 7 months, animals were housed individually in standard cages on sawdust bedding in an air-conditioned room (about 20 °C). They were kept under a reversed 12/12 h light/dark cycle (lights on from 20.00 to 08.00) and had free access to food and water. Mice were housed and tested in the same room. A radio, which was playing softly, provided background noise in the room. The object location task and Y-maze spontaneous alterations were performed as previously described [20, 33].

### Brain tissue processing

Mice were sacrificed by intracardial perfusion using PBS and heparin solution for 10 min under deep pentobarbital anesthesia (200 mg/kg). Brains (*n* = 14/genotype) were removed and hippocampus and prefrontal cortex were dissected, snap-frozen in liquid nitrogen and stored at − 80 °C until further processing.

### Cell culture

Neuro2a (N2a) mouse neuroblastoma (CCL-131™, ATCC, Wesel, Germany) and HT22 mouse hippocampal cell lines (kind gift from Dr. Amalia Dolga, Rijksuniversiteit Groningen) [34, 35] were cultured in DMEM/F-12 supplemented with GlutaMAX™ (Gibco), 10% fetal bovine serum, and penicillin/streptomycin. For treatment with GEBR32a (European patent EP2907806A1) and/or amyloid-β_1-42_ (Aβ_1-42_; AS-24224, Eurogentec), compounds were dissolved in 100% dimethyl sulfoxide (DMSO) and diluted into culture medium at a final concentration of 0.1% DMSO.

### Brain PDE4 activity

PDE activity was measured using a radioactive cAMP hydrolysis assay as described previously [36]. Specific PDE4 activity was determined as pmol cAMP hydrolyzed/min/mg protein as the activity that was ablated by inclusion of 10 µM rolipram (PDE4 inhibitor). Background readings were determined in control tubes where no protein was added. Background values were subtracted before values were normalized to pmol cAMP hydrolyzed/min/mg protein.

### Quantitative PCR

RNA was extracted from hippocampal and prefrontal cortical tissue or HT22 cells (treated with 0.1% DMSO or 1 µM Aβ_1-42_ for 24 h) using standard TRIzol-chloroform procedure (TRIzol, Invitrogen). Subsequently, complementary DNA (cDNA) was synthesized using qScript™ cDNA SuperMix (QuantaBio) according to the manufacturer’s protocol. Quantitative PCR (qPCR) was performed using the primers listed in Table 1 in the Supplementary Material (.doc) in combination with SensiMix 2X (Bioline) according to the manufacturer’s protocol. All qPCR reactions were performed in duplicate using 12.5 ng sample cDNA and the LightCycler 480 (Roche). The reaction protocol consisted of a polymerase activation cycle (95 °C for 10 min), 40 amplification cycles (95 °C for 15 s, primer-specific annealing temperature (*T*_a_; Supplementary Table 1) for 15 s and 72 °C for 15 s), followed by a melting curve cycle (95 °C for 5 s and 70 °C for 1 min) and cooling cycle (40 °C for 30 s). Raw expression data from the LightCycler 480 were analyzed using LinRegPCR to determine logarithmic fluorescence values at cycle zero (Log10 start fluorescence) [37]. qPCR reactions that did not show fluorescent signal amplification were excluded from further analysis. Logarithmic fluorescence values were normalized against the average expression of the reference genes that showed most stable expression based on geNorm criteria (i.e., Ywhaz and Ppia for hippocampus; Ywhaz and 18 s for frontal cortex; Ppia for HT22) [38]. Normalized data were used for statistical analysis.

### Genetic knockdown of PDE4D isoforms

Single guide RNAs (gRNA) targeted against murine *Pde4d* isoform-specific DNA sequences (Table 2 in Supplementary Material,.doc) were designed with the Zhang‐lab online webtool (http://crispr.mit.edu). gRNAs were checked in BLAST for target specificity and cloned as annealed oligos into the pSpCas9(BB)‐2A‐GFP (PX458) vector (kindly provided by Feng Zhang; Addgene plasmid #48,138; http://n2t.net/addgene:48138; RRID:Addgene_48138) as described previously [39]. Briefly, purchased *E. coli* (Stabl) expressing the PX458 vectors were cultured in LB with 100 ng/μl ampicillin. PX458 vectors were isolated and purified using a NucleoSpin Plasmid EasyPure kit (Macherey–Nagel). Purified DNA was quantified using a NanoDrop™ 2000 Spectrophotometer (ThermoFisher) and used for restriction using the FastDigest BpiI (IIs class) restriction enzyme (FD1014, Thermo Scientific). Digestion was validated by means of agarose gel electrophoresis, and cut vector was excised from the agarose gel using a NucleoSpin Gel and PCR Clean-up kit (Macherey–Nagel). Annealed gRNA oligos were ligated into the restricted and purified PX458 vector using T4 DNA Ligase (EL0014, Thermo Scientific). Correct incorporation of the gRNA and vector integrity were validated by means of Sanger sequencing and restriction analysis. One-Shot Stbl3 chemically competent *E. coli* (Invitrogen) was transformed with the ligated vectors by means of heat shock and then cultured in ampicillin-containing LB agar plates for subsequent colony selection. Colonies were picked and cultured, followed by vector isolation and purification using a NucleoBond Xtra Midi EF kit (Macherey–Nagel, Düren, Germany). Purified PX458 containing the gRNAs complementary to PDE4D isoform-specific sequences was used to transfect HT22 cells using NeuroMag reagent (Oz Biosciences). Unrestricted vector with a short scrambled gRNA sequence was used as a negative control vector. The expected frameshift frequencies upon CRISPR-induced DNA cuts (Table 2 in Supplementary Material,.doc) were calculated using the inDelphi machine-learning algorithm established by David K. Gifford, Jonathan Yee-Ting Hsu and Max Walt Shen [40]. Following the functional experiments, selections of sgRNAs were validated for their effective targeting of the predicted site. DNA from HT22 cells was isolated with the DNeasy Blood & Tissue kit (Qiagen) according to manufacturer’s instructions. A cell-free cleavage assay was performed using the Guide-It sgRNA In Vitro Transcription and Screening System (Takara Bio) according to manufacturer’s instructions. Primer sequences and results are described in Supplementary Table 3, Supplementary Table 4, and Supplementary Fig. 5.

### Western blotting

Mouse hippocampal (HIP) and prefrontal cortex (PFC) tissues were homogenized and reconstituted in lysis buffer (25 mM Tris–HCl, 150 mM NaCl, 0.5% sodium deoxycholate, 1% NP-40, 1 mM DTT, 0.1% SDS; pH 7.5), supplemented with complete protease inhibitor and PhosSTOP phosphatase inhibitor (Roche). Protein extracts were diluted in SDS sample buffer (10% SDS, 300 mM Tris–HCl, 0.05% bromothymol blue, 10% β-mercaptoethanol) and combined to create three replicates of 12.5 µg total protein per condition per tissue type. Cell extracts were resolved in 4–12% gradient SDS-PAGE and transferred onto nitrocellulose membranes (Bio-Rad Laboratories). Membranes were blocked in TBS-based Intercept Blocking Buffer (Li-Cor) for 1 h at room temperature, followed by incubation in Total Protein stain (Li-Cor, 926–11,011) to confirm appropriate transfer. Membranes were then de-stained as per manufacturer’s instructions (Li-Cor) and incubated overnight in rabbit anti-phosphoCREB (Ser133) (1:1000, #9191, Cell Signaling Technology), rabbit anti-CREB (1:1000, #9192, Cell Signaling Technology), rabbit anti-PKA phospho-substrate (1:1000, # 9621, Cell Signaling Technology), rabbit anti-Pan PDE4D (1:3000, Abcam, ab171749) and mouse anti-GAPHD (1:3000, Abcam, ab8245) at 4 °C, and subsequently incubated with donkey anti-rabbit IRDye 800 nm (1:10.000, Li-Cor, 926–33,213) and donkey anti-mouse IRDye 700 nm (1:10.000, Li-Cor, 926–68,072) for 1 h at room temperature.

For western blots of N2a cell extracts, cells were lysed using 3T3 lysis buffer (25 mM HEPES, 25 mM EDTA, 50 mM NaCl, 50 mM NAF, 30 mM sodium pyrophosphate, 10% glycerol, 1% Triton X; pH 7.5) supplemented with protease inhibitor mix and PhosSTOP phosphatase inhibitor (Roche). Cell extracts (12.5 µg total protein) were resolved in 10% SDS–polyacrylamide gel electrophoresis and then transferred onto nitrocellulose membranes (Bio-Rad Laboratories). The membranes were blocked (50% Odyssey blocking buffer in PBS; Li-Cor, Lincoln, NE) for 1 h at room temperature, followed by overnight incubation rabbit anti-phosphoCREB (Ser133) (1:1000, #9198, Cell Signaling Technology), mouse anti-CREB (1:1000, #9104, Cell Signaling Technology) and mouse anti-GAPDH (1:1,000,000, #10R-G109A; Fitzgerald Industries, Acton, MA) at 4 °C. Membranes were subsequently incubated with goat anti-rabbit IRDye 800 (1:10.000, Li-Cor, 926–32,211) and donkey anti-mouse IRDye 680 (1:10.000, Li-Cor, 926–68,072) for 1 h at room temperature. All immunoblots were visualized using the Odyssey CLx scanner, and protein bands were quantified using ImageJ by means of densitometry [41].

### Neuronal morphology assessment

To assess neuronal morphology of N2a and HT22 cells, cells were seeded in 24- or 48-well plates to be treated with GEBR32a and/or Aβ_1-42_ or to be transfected. N2a cells were treated with DMSO or 0.1–1.0 µM GEBR32a for 48 h (*n* = 6/condition). HT22 cells were incubated with DMSO (*n* = 12/condition) or 0.01–1.0 µM GEBR32a (*n* = 6/condition). In another set of experiments, HT22 cells were treated with 0.5–1.0 µM Aβ_1-42_ alone and GEBR32a (1 µM) and Aβ_1-42_ (1 µM) and incubated for 24 h (*n* = 6/condition). For transfection experiments, HT22 cells were cultivated for 48 h upon transfection or exposed to 1 µM Aβ_1-42_ for 24 h, one-day post-transfection (*n* = 9–12/condition). To visualize and quantify neuronal morphology, the Neurite Outgrowth Staining Kit (A15001, Invitrogen) was used to fix and membrane-stain the cells according to the manufacturer’s protocol. Pictures (20 × magnification) were taken with an Olympus IX81 inverted microscope connected to an ORCA-fusion digital CMOS camera (C14440-20UP, Hamamatsu) using the MicroManager open source software [42, 43]. Per well, three images were captured and used for neurite outgrowth analysis by the NeuronJ plugin for ImageJ [41, 44]. Average neurite length per condition was normalized against the control conditions (i.e., DMSO-treated or control-transfected cells).

### cAMP determination in cultured cells

To evaluate the effect of PDE4D inhibition by GEBR32a on global intracellular cAMP levels, N2a and HT22 cells were seeded in 12-well plates at 10^6^ cells per well. Cells were treated with DMSO or GEBR32a 0.01–3 µM (n ≥ 4/condition) and 1 µM of the adenylyl cyclase activator forskolin (MedChemExpress, HY-15371) for 1 h and subsequently lysated in 0.1 M HCl. Concentrations of cAMP were measured using the Cyclic AMP ELISA Kit (Cayman Chemical Company, 581,001) according to manufacturer’s instructions. Results are depicted as fold change over DMSO control.

### Immunocytochemistry

To investigate PDE4D protein localization, HT22 cells were seeded on 12 mm glass coverslips (VWR, 631–1577) coated with 100 μg/mL Poly-l-Ornithine (Sigma, P4957) and 1 μg/mL laminin (Sigma, L2020) and grown for 24 h. After fixation in 4% paraformaldehyde, cells were permeabilized using 0.1% Triton X-100. After blocking with 10% BSA for 1 h, cells were incubated with rabbit anti-PDE4D (1:250; ab14613, Abcam) overnight at 4 °C. Then, cells were incubated with goat anti-rabbit Alexa647 (1:250; Invitrogen) for 1 h at room temperature. Finally, nuclei were counterstained with Hoechst (1:500; Sigma).

For immunocytochemistry following transfection experiments in HT22, the same protocol was used except for the antibodies used. Mouse anti-FLAG primary antibodies (1:1000; M2 clone, Sigma-Aldrich) and donkey anti-mouse Alexa488-conjugated secondary antibody (1:250; Invitrogen) were used to determine which cells were successfully transfected and expressed the FLAG-encoding PX458 plasmid. PDE4D localization was imaged after mounting the coverslips on microscope glasses using a disk spinning unit (DSU) microscope (Olympus). Morphology assessment of transfected, FLAG-positive HT22 cells was performed as described above.

### Gene ontology (GO) term enrichment analysis

Determining the involvement of PDE4D in specific biological processes was approached by examining whether known PDE4D interaction proteins are enriched in these processes. The list of known PDE4D interactors was retrieved from BioGRID [45] and used for gene ontology (GO) term enrichment analysis using ToppGene Suite with default settings (https://toppgene.cchmc.org/) [46]. The complete GO analysis output can be found in Supplementary Table 3 (.xls).

### Statistical analysis

For the OLT and Y-maze behavioral tasks, memory performance of WT and APPswe/PS1dE9 was assessed statistically by performing two-tailed paired *t* tests between genotypes and comparing performance of each genotype against chance performance (i.e., 0 for OLT and 50% for Y-maze). Likewise, normalized qPCR, PDE4 activity, and WB values were used to compare expression/activity differences between genotypes by means of two-tailed t tests. For in vitro studies on neurite length, CREB phosphorylation, and cAMP concentration, one-way ANOVAs were performed with Dunnett’s post hoc t tests using the DMSO condition as control. Differences in PDE4D isoform mRNA expression between DMSO- and Aβ-treated HT22 cells were investigated by means of two-tailed t tests. The effect of genetic knockdown of PDE4D isoforms on neuronal morphology was analyzed by means of one-way ANOVA of normalized neurite lengths followed by Dunnett’s post hoc t tests. The effect of genetic knockdown of PDE4D isoforms in combination with Aβ exposure was compared to control-transfected conditions with and without exposure to Aβ by means of one-way ANOVA followed by Dunnett’s post hoc t tests.

## Results

### Impaired spatial memory and altered PDE4D-mediated cAMP-PKA-CREB signaling in female APP/PS1 mice

**Fig. 1:**
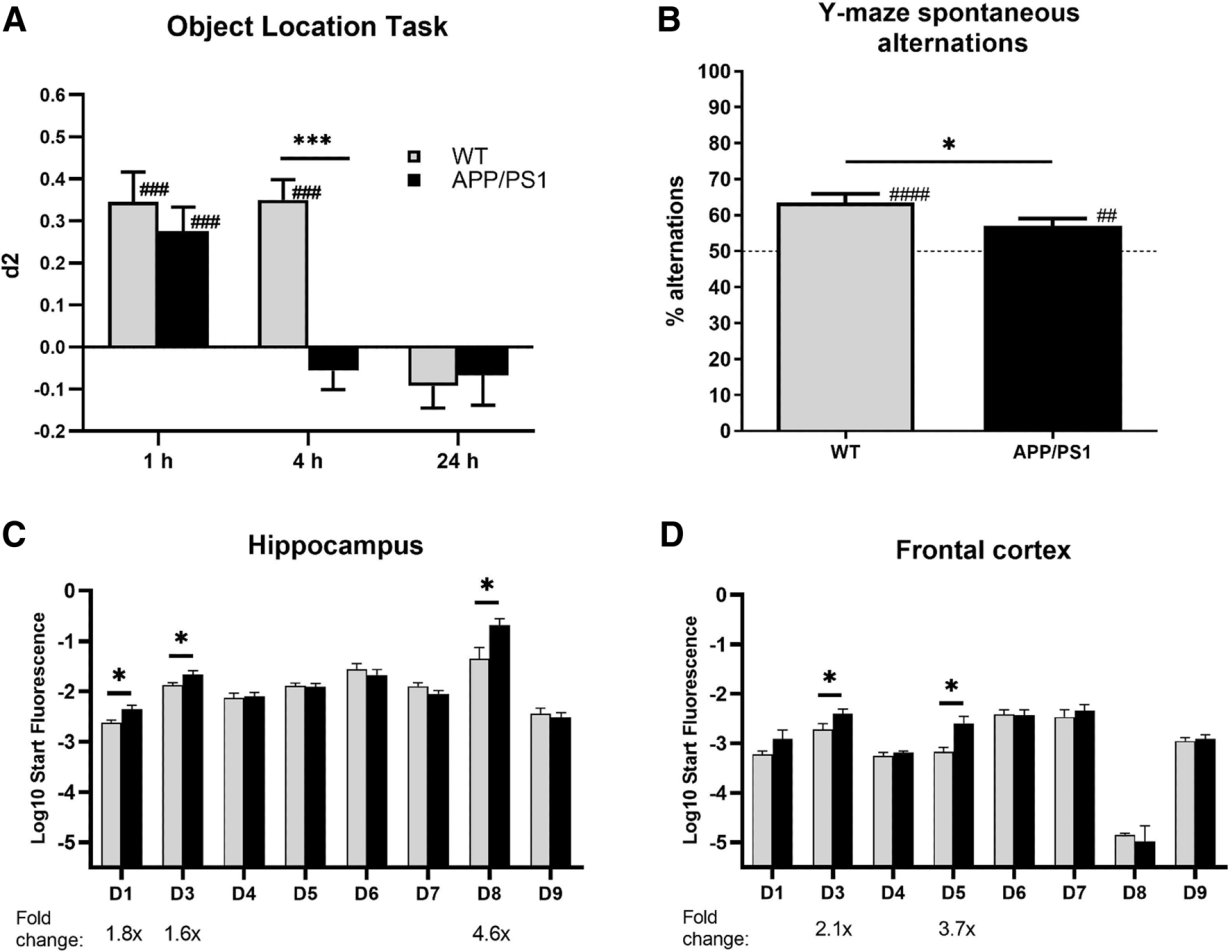
Changes in memory performance and PDE4D-mediated cAMP-PKA-CREB signaling in female APP/PS1 mice. **A** Spatial memory performance of female WT and APP/PS1 mice in the object location task. Using a 4-h inter-trial interval revealed impaired memory performance in APP/PS1, but not wild-type (WT) mice (***P < 0.001, two-tailed t-test comparing genotypes; ^###^*P* < 0.001, paired two-tailed t test against 0; *n* = 18–22/genotype). **B** Spatial memory performance in the Y-maze spontaneous alterations task. Intact memory, i.e., higher than chance (50%), was found for both genotypes and significantly higher memory performance in WT versus APP/PS1 mice (*P < 0.05, two-tailed *t*-test; ^##^*P* < 0.01, ^###^*P* < 0.001, paired two-tailed *t*-test against 50%; *n* = 18–22/genotype). C-D) PDE4D isoform mRNA expression in hippocampus and frontal cortex of WT and APP/PS1 mice (*n* = 7–8/genotype). Significantly increased expression of specific isoforms was measured in APP/PS1 versus WT mice (*P < 0.05, two-tailed t test)

Spatial memory assessment in the OLT and Y-maze spontaneous alterations task demonstrated that APP/PS1 mice were cognitively impaired compared to WT mice. More specifically, in the OLT, both genotypes showed intact memory performance using a 1-h inter-trial interval (Fig. 1A; ###*P* < 0.001 two-tailed t test against 0). Using a 4-h interval, WT mice showed intact spatial memory, while APP/PS1 did not (###*P* < 0.001 paired two-tailed *t* test against 0). In addition, APP/PS1 mice performed significantly worse than WT mice (Fig. 1A; ***P < 0.001 two-tailed t-test). Similarly, while both genotypes performed significantly better than chance level in the Y-maze spontaneous alterations task (Fig. 1B; ^##^*P* < 0.01, ^###^*P* < 0.001, paired two-tailed t test against 50%), APP/PS1 mice performed worse than WT mice (*P < 0.05, two-tailed *t* test).

**Fig. 2:**
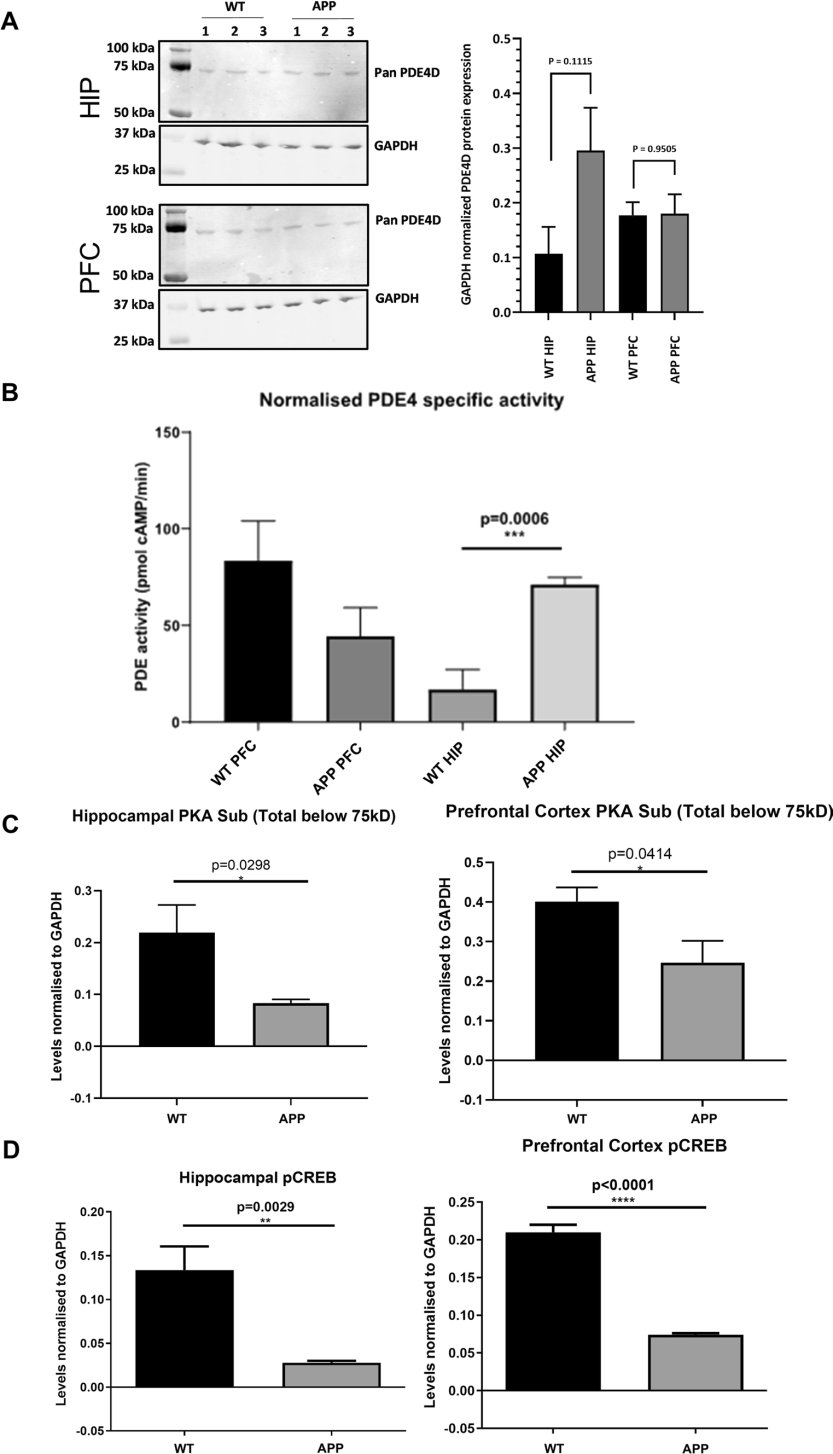
Changes in PDE4D protein expression, PDE4 activity and cAMP-PKA-CREB signaling in female APP/PS1 mice. **A** PDE4D protein expression in hippocampus and cortex of female WT and APP/PS1 mice (*n* = 3/genotype). **B** Specific PDE4 activity in homogenates of prefrontal cortex (PFC) and hippocampal (HIP) tissue excised from APP/PS1 and WT mice normalized for amount of protein (*n* = 3/genotype). **C** Densitometric quantification of western blots measuring phospho-PKA substrates normalized to GAPDH in WT versus APP/PS1 mice (*n* = 6/genotype). **D** Densitometric quantification of western blots measuring phosphorylated CREB (pCREB) normalized to GAPDH in WT versus APP/PS1 mice (n = 6/genotype, *P < 0.05, **P < 0.01, ****P < 0.0001, two-tailed t-test). Data are presented as mean + SEM. Western blot scans are provided in the Supplementary Material (.doc)

After observing the memory deficits in APP/PS1 mice, hippocampal and frontal cortical tissue of these animals was used for biochemical assessments to investigate potentially associated alterations in PDE4D-mediated cAMP-PKA-CREB signaling. PDE4D mRNA expression of specific isoforms was found to be higher in APP/PS1 in both brain regions. In the hippocampus, PDE4D1, -D3, and -D8 expression was 1.6- to 4.6-fold higher, while PDE4D3 and -D5 were 2.1- to 3.7-fold upregulated in the frontal cortex (Fig. 1C, D; *P < 0.05, two-tailed t test). At the protein level, general PDE4D expression showed a non-significant increase in the APP/PS1 hippocampus, which was not observed in the frontal cortex (Fig. 2A). PDE4 activity was found to be threefold higher in the hippocampus, but not in the frontal cortex, of APP/PS1 compared to WT mice (Fig. 2B; ***P < 0.001, two-tailed t test). Moreover, assessment of protein expression of phosphorylated PKA substrates and phosphorylated CREB was found to be significantly lower in the hippocampus and frontal cortex of APP/PS1 mice (Fig. 2C, D; *P < 0.05, **P < 0.01; ***P < 0.001, ****P < 0.0001).

### Pharmacological PDE4D inhibition increases CREB phosphorylation and neurite length in N2a cells

**Fig. 3:**
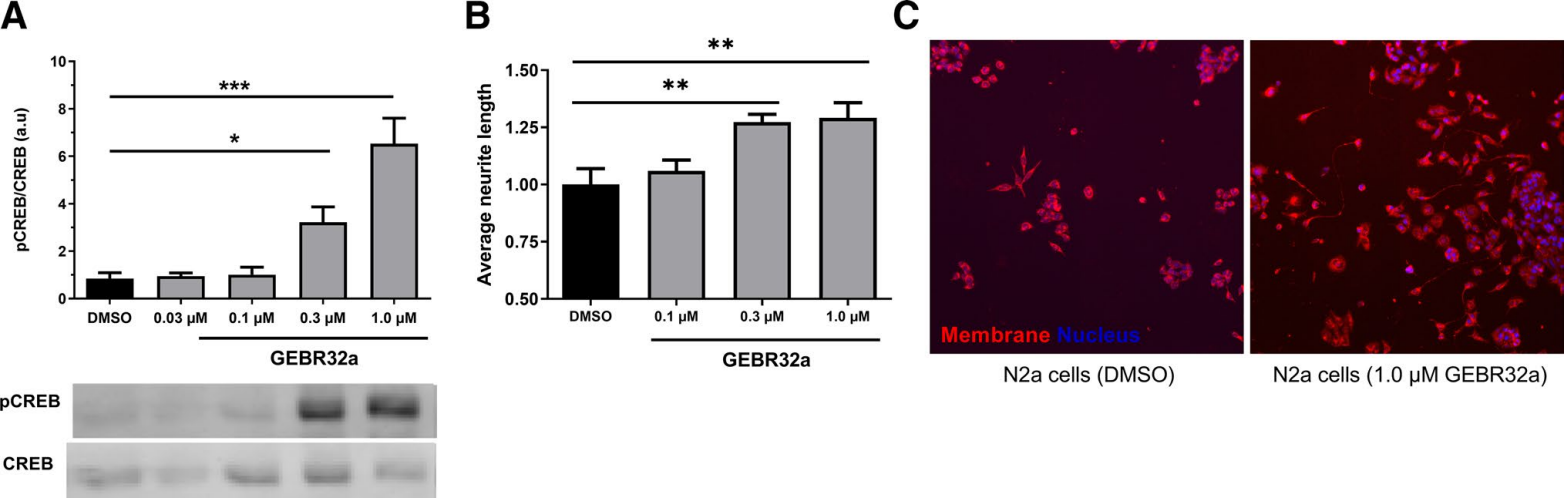
PDE4D inhibition dose-dependently increased CREB phosphorylation and neurite length in N2a cells. **A** Phosphorylated CREB (pCREB) to total CREB ratios in N2a cells upon treatment with 0.03–1.0 µM GEBR32a for 1 h (Dunnett's post-hoc: *P < 0.05, ***P < 0.001; *n* = 6/condition). Representative blot scans are shown under the graph. **B** Average neurite after incubation with 0.1–1.0 µM GEBR32a for 48 h in N2a cells (Dunnett’s post-hoc: **P < 0.01; *n* = 6/condition). **C** Exemplary microscope images indicating N2a neurites in the DMSO (left) and 1.0 µM GEBR32a (right) conditions that were visualized for subsequent tracing. Data is presented as mean + SEM

To validate the involvement of PDE4D in cAMP-PKA-CREB signaling and neuronal plasticity, N2a cells were treated with the PDE4D-selective inhibitor GEBR32a. After 1 h incubation with GEBR32a (0.03–1.0 µM), phosphorylated CREB (pCREB) and total CREB levels were measured by means of western blotting. One-way ANOVA revealed significant differences in normalized pCREB/CREB ratio between conditions (*F*(5,30) = 17.83, *P* < 0.0001). Compared to the DMSO condition, significant increases in pCREB/CREB ratio were found for 0.3 and 1.0 µM GEBR32a (*P < 0.05, **P < 0.05, Dunnett’s post-hoc t-tests; Fig. 3A). Moreover, upon 1 h treatment with 3 μM GEBR32a, cAMP levels in N2a cells (and likewise HT22 cells) can increase up to fivefold compared to the DMSO control (Dunnett’s post-hoc t-tests; Supplementary Fig. 4).

Similarly, N2a cells were treated with GEBR32a to assess the effects of PDE4D inhibition on neuronal morphology by measuring neurite lengths. After 48 h incubation with GEBR32a (0.1–1.0 µM) neurite lengths were measured. One-way ANOVA revealed significant differences in the average neurite length between conditions (*F*(3,20) = 6.886, *P* < 0.01). Compared to the DMSO condition, significant increases in average neurite length were found for 0.3 and 1.0 µM GEBR32a (**P < 0.01, Dunnett’s post-hoc *t*-tests; Fig. 3B, C). These findings indicate that, in the N2a cell line, CREB phosphorylation and neurite elongation are stimulated by GEBR32a-induced PDE4D inhibition at 1 h and 48 h, respectively.

### Pharmacological PDE4D inhibition induces longer neurites in mouse hippocampal cells

**Fig. 4:**
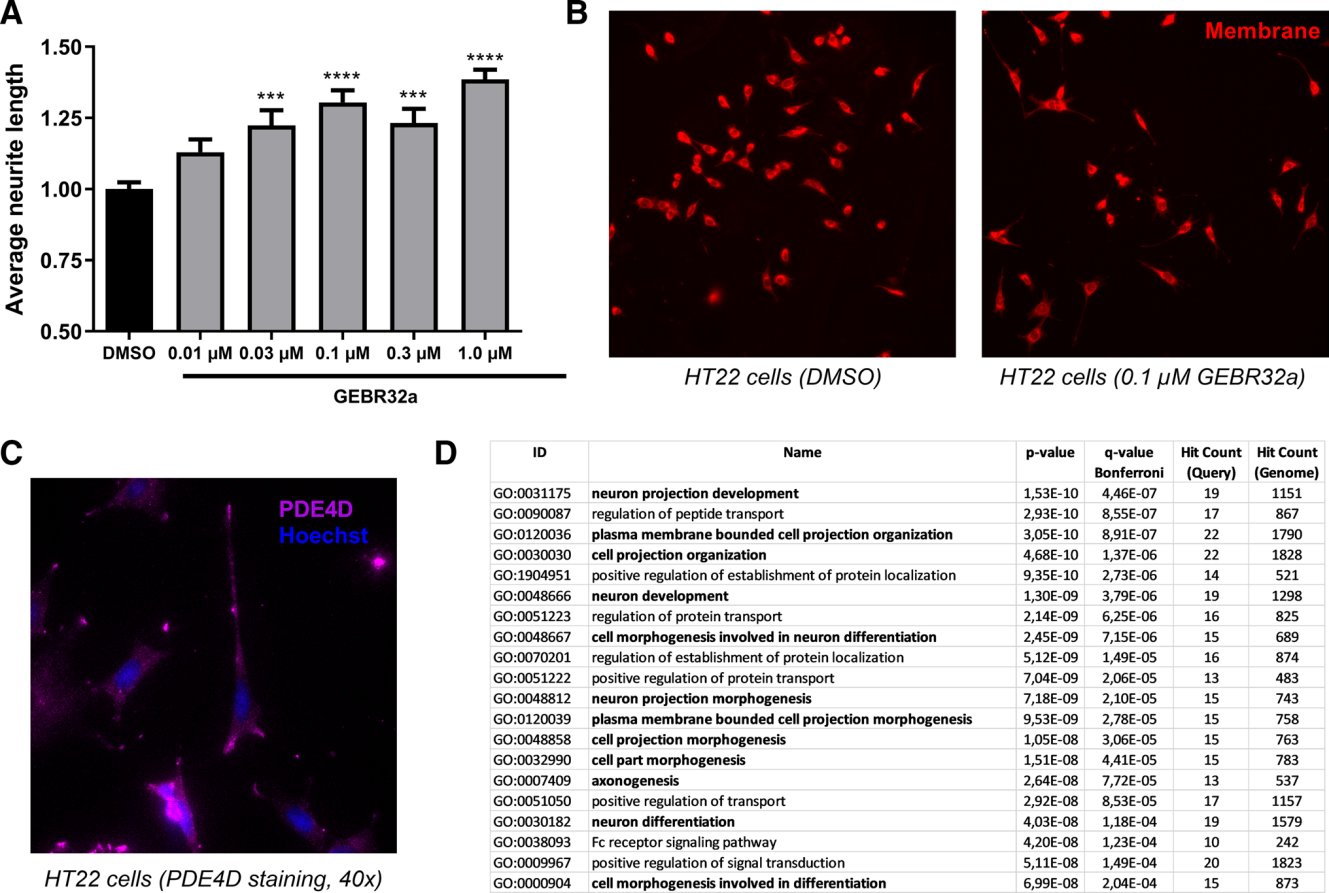
PDE4D regulates neuronal morphology, PDE4D localization, and gene ontology term enrichment of PDE4D interaction proteins. **A** Average neurite length after incubation of HT22 cells with 0.01–1.0 µM GEBR32a for 48 h (Dunnett’s post hoc: ***P < 0.001, ****P < 0.0001; *n* = 6–12/condition). **B** Representative microscope images indicating HT22 neurites in the DMSO (left) and 0.1 µM GEBR32a (right) conditions that were visualized for subsequent tracing. **C** Representative HT22 immunofluorescence image showing the predominant localization of PDE4D in neurites and their growth cones. **D** Gene ontology (GO) term enrichment analysis of biological processes of PDE4D interaction proteins (retrieved from BioGRID [45]) showing enrichment for processes related to (neuronal) morphology regulation (in bold). Top twenty terms are shown, the entire list is provided in Supplementary Table 3 (.xls). Data are presented as mean + SEM

Next, complementing the findings in the mouse neuroblastoma N2a cells, we sought to identify the role of PDE4D in regulating neuronal morphology in the mouse hippocampal HT22 cell line to better approximate the in vivo context in which we found changes in hippocampal PDE4D-mediated cAMP-PKA-CREB signaling (Figs. 1 and 2). Specifically, HT22 cells were incubated with 0.01–1.0 µM GEBR32a for 48 h to assess the effects of pharmacological PDE4D inhibition on neuronal morphology. One-way ANOVA revealed significant differences in the average neurite length between conditions (*F*(5,36) = 14.60, *P* < 0.001). Compared to control samples, significant increases in average neurite length were found in cells treated with 0.03–1.0 µM GEBR32a (Fig. 4A, B; ***P < 0.001, ****P < 0.0001, Dunnett’s post hoc t tests).

As inhibition of PDE4D significantly increased neurite length, we investigated whether PDE4D is localized in or near neurite growth cones to locally regulate cAMP signaling. Based on immunocytochemistry, it was found that PDE4D is expressed in the neurite growth cones of HT22 cells (Fig. 4C). Additional support for a role of PDE4D in regulating neuronal morphology was found by investigating the shared biological processes of known PDE4D interaction proteins. The list of known PDE4D interaction proteins was retrieved from BioGRID [45] and subjected to gene ontology (GO) enrichment analysis using ToppGene Suite [46]. PDE4D-interacting proteins were found to be significantly enriched in biological processes regulating (neuronal) morphology (Fig. 4D). More specifically, ‘neuron project development’ (GO:0,031,175) was found to be most significantly enriched. Through interaction with the proteins enriched in these processes, PDE4D is likely to be optimally localized to regulate neuronal morphology. The complete GO analysis including interaction proteins per biological process can be found in Supplementary Table 3 (.xls).

### Pharmacological PDE4D inhibition protects against Aβ_1-42_-induced reductions in neurite length

**Fig. 5:**
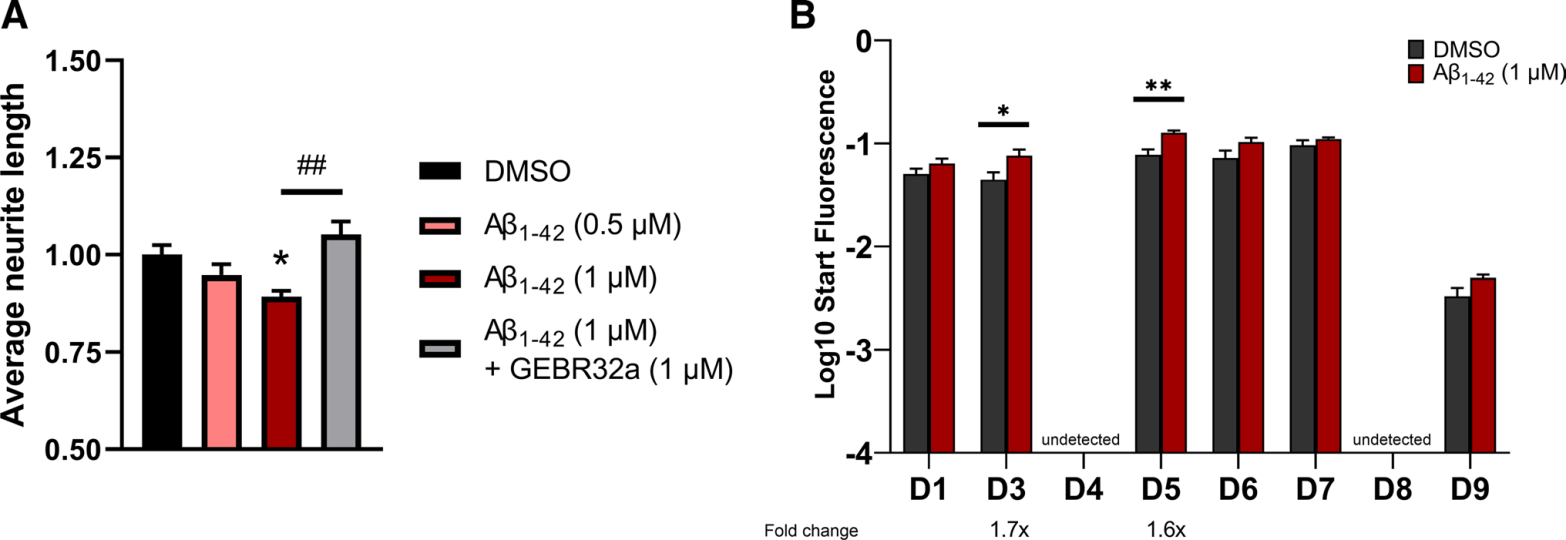
Aβ_1-42_ exposure reduced the average neurite length in HT22 cells and increased isoform-specific PDE4D expression. **A** Average neurite length after incubation of HT22 cells with 0.5–1.0 µM Aβ_1-42_ or GEBR32a and Aβ_1-42_ (both 1 µM) for 24 h (Dunnett’s post hoc: *P < 0.05; two-tailed *t*-test: ##*P* < 0.01; n = 6/condition). **B** PDE4D isoform mRNA expression after 1 µM Aβ_1-42_ exposure for 24 h in HT22 (*P < 0.05, **P < 0.01, two-tailed *t* test; n = 6/condition). Data are presented as mean + SEM

Since APP/PS1 mice showed impaired spatial memory performance and altered cAMP-PKA-CREB signaling as a result of excessive Aβ production, we subsequently examined whether Aβ_1-42_ exposure negatively impacts neurite growth in HT22 cells and whether PDE4D inhibition can protect against these effects. HT22 cells were incubated for 24 h with 0.5–1.0 µM Aβ_1-42_ or a combination of Aβ_1-42_ and GEBR32a (both 1 µM). One-way ANOVA revealed significant differences in the average neurite length between conditions (*F*(3,20) = 6.932, *P* < 0.01). Compared to the DMSO condition, a significantly decreased average neurite length was found for 1.0 µM Aβ_1-42_ (Fig. 5A; *P < 0.05, Dunnett’s post hoc t tests). Combined incubation with Aβ_1-42_ and GEBR32a was able to prevent this Aβ_1-42_-induced neurite length reduction (Fig. 5A; ^##^*P* < 0.01, two-tailed *t*-test). PDE4D expression measurement indicated that, concurrent with reducing the HT22 average neurite length, 1 µM Aβ_1-42_ increases mRNA expression of the specific isoforms PDE4D3 and -D5 (Fig. 5B; *P < 0.05, **P < 0.01, two-tailed *t* test). It should be noted that PDE4D4 and PDE4D8 mRNA could not be detected in HT22 cells.

### Neurite growth is regulated by long PDE4D isoforms both in absence and presence of Aβ_1-42_

**Fig. 6:**
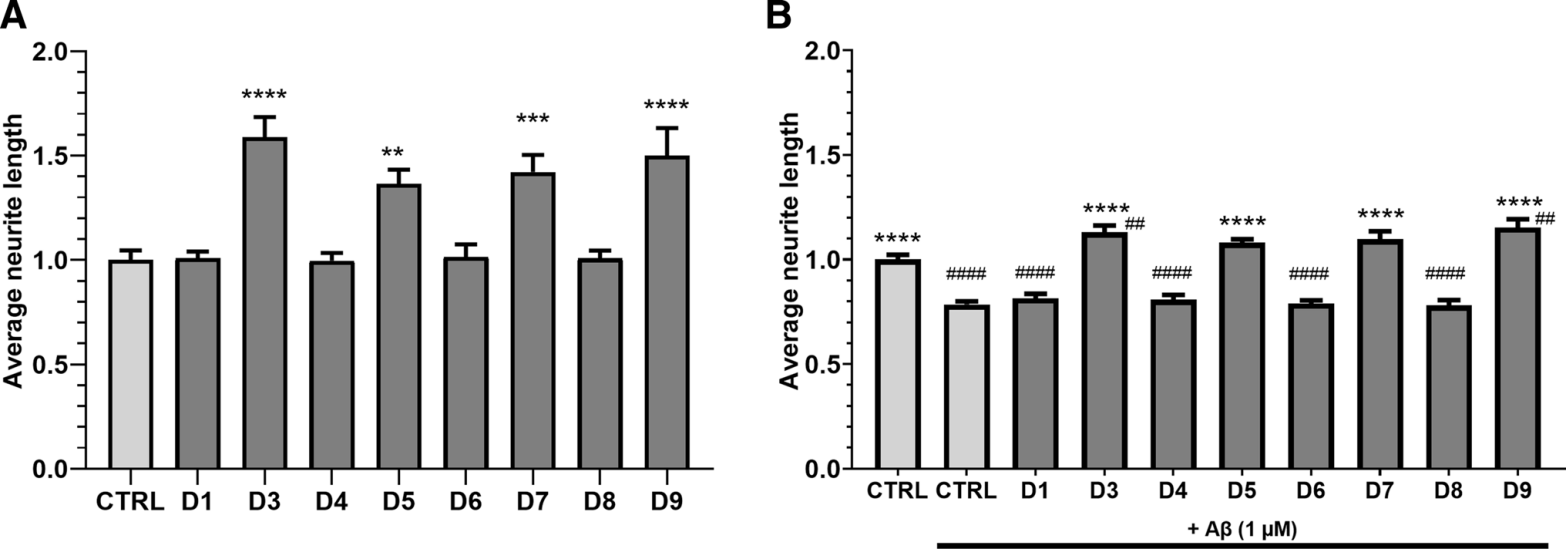
Knockdown of specific PDE4D isoforms increases neurite length and protects against Aβ_1-42_-induced neurite length reduction. HT22 cells were transfected with CRISPR-Cas9 vectors to genetically knock down specific PDE4D isoforms. **A** Average neurite lengths upon PDE4D isoform knockdown 48 h after transfection (**P < 0.01, ***P < 0.001, ****P < 0.0001, Dunnett’s post hoc *t* tests; *n* = 9–12/condition). **B** Average neurite lengths upon PDE4D isoform knockdown 48 h after transfection with 1 µM Aβ_1-42_ exposure during the last 24 h (****P < 0.0001, Dunnett’s post hoc *t* tests compared to control with Aβ_1-42_ exposure; *n* = 9/condition). Comparisons against control transfection without Aβ_1-42_ exposure are shown by hashes (^##^*P* < 0.01, ^###^#*P* < 0.0001, Dunnett’s post hoc t tests). Data are presented as mean + SEM

PDE4D isoforms seem to be differentially expressed upon Aβ_1-42_ exposure both in vivo (Fig. 1C, D) and in vitro (Fig. 5B), and isoforms are known to localize to distinct cellular compartments to regulate specific processes [2]. As non-specific inhibition of all PDE4D isoforms may cause severe adverse effects, we investigated whether specific PDE4D isoforms regulate neurite growth in HT22 cells and could serve as a more specific therapeutic target for AD treatment. Individual PDE4D isoforms were genetically knocked down in HT22 cells by transfection with CRISPR-Cas9 vectors (transfection efficiency of approximately 50%), after which average neurite length was measured. 48 h after transfection, one-way ANOVA revealed significant differences in the average neurite length between conditions (*F*(8,75) = 12.36, *P* < 0.0001). Compared to the control condition, 1.36- to 1.59-fold increased average neurite lengths were found for knockdown of PDE4D3, -D5, -D7, and -D9 (Fig. 6A; **P < 0.01, ***P < 0.001, ****P < 0.0001, Dunnett’s post hoc *t* tests).

Next, it was examined whether PDE4D isoform knockdown could prevent Aβ_1-42_-induced reductions in average neurite length. To this purpose, PDE4D isoforms were knocked down and cells were exposed to 1 µM Aβ_1-42_ for 24 h one-day post-transfection after which average neurite lengths were measured. One-way ANOVA revealed significant differences in the average neurite length between conditions (*F*(8,68) = 36.34, *P* < 0.0001). In the presence of Aβ_1-42_, knockdown of PDE4D3, -D5, -D7, and -D9 increased average neurite length by 1.38- to 1.46-fold. Increased average neurite lengths were found when compared to the control condition with Aβ_1-42_ (****P < 0.0001, Dunnett’s post hoc *t* tests; Fig. 6B). Moreover, in the absence of Aβ_1-42_, average neurite length upon control transfection was significantly higher than upon control transfection with Aβ_1-42_ (****P < 0.0001, Dunnett’s post hoc t tests; Fig. 6B). Compared to the control condition without Aβ_1-42_, PDE4D3 and -D9 knockdown countered the effect of Aβ_1-42_ and was associated with significantly increased average neurite length (^##^*P* < 0.01, Dunnett’s post hoc *t* tests, Fig. 6B). Knockdown of PDE4D1, -D4, -D6, and -D8 with exposure to Aβ_1-42_ led to significantly lower average neurite lengths compared to the control condition without Aβ_1-42_ exposure (*P* < 0.0001, Dunnett’s post hoc t test, not shown in the graph).

## Discussion

cAMP-PKA-CREB signaling has been well established as a pathway that is crucial for synaptic plasticity and memory consolidation [1]. Moreover, impaired memory consolidation and cognitive deficits in AD have been associated with deficient cAMP-PKA-CREB signaling [47]. Inhibition of the cAMP-degrading PDE4D enzymes has been found to restore memory functioning in transgenic AD mice [20, 21]. Despite this therapeutic potential, non-specific PDE4D inhibition has been associated with severe adverse effects (e.g., nausea and vomiting), which has hampered clinical progression of PDE4(D) inhibitors [3, 13]. Remarkably, PDE4D enzymes comprise multiple protein isoforms that show tissue- and cell type-specific expressions and regulate distinct intracellular processes [2, 13, 27]. Hence, treatment efficacy may be enhanced or maintained, while side effects are reduced, by specifically targeting those PDE4D isoforms that are involved in synaptic plasticity. Therefore, we investigated whether PDE4D isoforms are differentially expressed in transgenic AD mice and whether specific PDE4D isoforms control neurite growth as a measure of plasticity. Additionally, we examined whether PDE4D inhibition or individual PDE4D isoform knockdown could protect against Aβ-induced toxicity.

Cognitive phenotyping revealed spatial memory deficits in female APP/PS1 mice at 7 months of age. These findings replicate a prior study using the same genotypes and behavioral tasks [20]. In our follow-up biochemical assessment, it was demonstrated that CREB phosphorylation was reduced in the hippocampus and frontal cortex of APP/PS1 mice. Moreover, we measured mRNA expression increases for PDE4D1, -D3, and -D8 in the hippocampus and for PDE4D3 and -D5 in the frontal cortex of APP/PS1 mice. Recently, we also found the same PDE4D isoforms to show pathology-associated, increased expression in post-mortem human middle temporal gyrus tissue of AD patients [25]. In addition, the observation of increased PDE4D3 mRNA expression in the hippocampus of APP/PS1 mice corresponds to the fact that hippocampal injections of Aβ_1-42_ in wild-type mice caused elevated PDE4D(3) protein expression [48]. Here, we also show that PDE4 activity was increased in the hippocampus of APP/PS1 mice, which may be a result of increases in PDE4D3 expression. However, PDE4 activity is not correlated one-on-one with PDE4D activity as other PDE4 isoforms also contribute, possibly explaining why this increase in activity cannot be observed in PFC of APP/PS1 mice. While we report changes in PDE4D isoform mRNA expression, a non-significant trend of increase was found for PDE4D protein expression in the hippocampus of APP/PS1 mice using an antibody that is not isoform-specific. Multiple isoforms (e.g., PDE4D3 and PDE4D8) would migrate identically on SDS-PAGE and could therefore not be distinguished [49]. However, our observations are in accordance with previous reports showing increases in hippocampal PDE4D protein expression in transgenic Alzheimer mice and mice exposed to exogenous Aβ [12, 50]. Whether protein levels of specific PDE4D isoforms are upregulated while others are unaffected or are downregulated remains to be determined. Most importantly, the in vivo experiment was conducted to provide proof-of-concept of the change in PDE4D isoform signature during AD-related cognitive decline. Results are similar to the results from our human AD post-mortem study where we included both female and male samples, yielding comparable results [25]. Hence, the finding in these female mice provided a step toward the in vitro pharmacological inhibition and genetic editing in mouse cells. However, the use of female mice only might serve as a limitation.

When examining whether PDE4D inhibition can stimulate cAMP-PKA-CREB signaling, we found that the PDE4D-selective inhibitor GEBR32a significantly increased CREB phosphorylation in vitro, which is in accordance with previous studies using PDE4 and PDE4D inhibitors [17, 51]. Moreover,GEBR32a at a concentration of 3 μM substantially increased global intracellular cAMP levels in both HT22 and N2a cells. This is in line with the IC_50_ of the compound (2.43 μM) [22]. Furthermore, pharmacological PDE4D inhibition induced a dose-dependent increase in neurite length in both N2a and HT22 cells. The role of PDE4D in regulating neurite outgrowth was supported by the localization of PDE4D protein in growth cones and the fact that many of known PDE4D-interacting proteins are involved in neuron projection development. As PDE4D was also found to localize to microtubules in neurons of the macaque prefrontal cortex [52], there is evidence that PDE4D also regulates neuronal morphology in vivo. Pharmacological PDE4D inhibition by GEBR32a enhances neurite outgrowth in N2a and HT22 cells starting at a concentration of 0.03 μM. Supposing that global cellular cAMP levels only rise detectably with an ELISA upon 3 μM GEBR32a exposure, lower-dose initiated cAMP changes on a nanoscale level, which would require new high-end detection techniques, are likely sufficient to stimulate neurite growth, providing additional support for PDE4D-associated low-cAMP compartments near growth cones [53].

Early studies already showed that Aβ exposure can cause neurite degeneration in hippocampal neurons [54]. The HT22 mouse hippocampal neuronal cell line we used here also showed reduced neurite length when exposed to 1 µM Aβ_1-42_, which corresponds to the concentrations that were neurotoxic to HT22 cells in prior studies [55]. Pharmacological PDE4D inhibition protected against this Aβ_1-42_-induced neurite length reduction.

However, as PDE4D inhibition has been associated with severe adverse effects, which may be due to central and/or peripheral actions, targeting of specific PDE4D isoforms could be therapeutically more effective or enable maintenance of efficacy while improving safety. The PDE4D isoforms (PDE4D1-9) are categorized into long, short, and supershort isoforms, which is based on the inclusion of specific exons and the isoform’s eventual protein sequence. The differences in sequence enable for isoform-specific activity modulation by post-translational modifications and for specific intracellular targeting to regulate specific processes [13]. Hence, we sought to investigate which PDE4D isoforms regulate neurite growth and convey the protective effect of PDE4D inhibition against Aβ_1-42_ exposure. We found that genetic ablation of the long PDE4D isoforms PDE4D3, -D5, -D7, and -D9 increased average neurite length both in absence and in presence of the neurotoxic Aβ_1-42_ concentration. Genetic knockdown of the other long isoforms, PDE4D4 and PDE4D8, had no effect on neurite length, which can be explained by the observation that these forms are not expressed in HT22 cells. Since PDE4D8 was expressed more in APP/PS1 hippocampus, this may be linked to expression in non-neuronal cell types (e.g., microglia, astrocytes and/or oligodendrocytes). Remarkably, the short PDE4D1 and supershort PDE4D6 isoform did not affect neurite growth upon genetic knockdown, which implies that neurite outgrowth is specifically regulated by long PDE4D isoforms. Complementary with our finding, it was recently published that knockdown of (super)short isoforms (i.e., PDE4D1 and PDE4D6) by the same CRISPR-constructs as used in this study results in an increase in oligodendrocyte precursor cell differentiation [56]. Thus, the constructs that did affect neuronal differentiation did not affect oligodendrocyte differentiation, and vice versa. This emphasizes the involvement of distinct PDE4D isoforms in distinct cell types. Of note, we validated that the sgRNAs for PDE4D1, PDE4D3, PDE4D6 and PDE4D9 were able to selectively induce a double stranded break at the desired site with high efficiency in a cell-free cleavage assay. Together, the transfection efficiency (50%), the predicted INDEL repair efficiency, and in vitro sgRNA cleavage data provide a proxy to estimate PDE4D isoform interference.

The question remains whether PDE4D long isoforms regulate the same cellular process or modulate different processes that eventually influence neurite outgrowth. For example, non-specific PDE4 inhibition was found to increase phosphorylation of vasodilator-stimulated phosphoprotein (VASP), a protein that is associated with the cytoskeleton [57], which is likely caused by PKA activation and will promote neurite elongation [28, 29, 58]. Since PDE4D localizes in HT22 neurite growth cones and long PDE4D isoforms appear to regulate neurite outgrowth, these long isoforms may control neuronal morphology by controlling PKA activity, and subsequent phosphorylation of specific targets, in neurite growth cones.

PDE4 subtypes and isoforms are known to localize to specific intracellular compartments [2, 26, 27, 59, 60]. Previous studies may provide insight into how PDE4D3, -D5, -D7, and -D9 influence cAMP signaling in regulating neurite growth. Most notably, PDE4D3 has been reported to bind perinuclear mAKAP, and the PDE4D3/mAKAP complex was found to support survival of and axon growth in neurons [61, 62]. Moreover, PDE4D3 can bind to the centrosome via AKAP9, thereby regulating cell cycle progression [63]. Thus, genetic knockdown of PDE4D3 likely promoted cAMP-PKA signaling in both the perinuclear and centrosomal regions, which may have halted cell cycle progression and promoted neurite growth. In addition, the increased perinuclear cAMP-PKA signaling may have stimulated PKA-mediated CREB phosphorylation, which subsequently could have promoted neuronal plasticity processes (e.g., neurite outgrowth).

By preferentially binding the scaffolding β-arrestin, PDE4D5 can be located in close vicinity of G-protein coupled receptors (GPCRs) to regulate cAMP levels at the site of synthesis near GPCR-stimulated adenylyl cyclase [26, 64, 65]. Additionally, PDE4D5 is located in the nucleus through interaction with AKAP95 and may therefore also be the specific isoform found in nuclei of cortical neurons in the macaque brain [52, 66]. Upon PDE4D5 knockdown, nuclear cAMP-PKA-CREB-mediated signaling and subsequent transcription may be stimulated while also cAMP levels are elevated near GPCRs to induce PKA-mediated phosphorylation of, for example, cytoskeletal proteins to promote neurite outgrowth. Cytoskeletal remodeling and neuronal motility are likely regulated by PDE4D5 species that are complexed with focal adhesion kinase (FAK) through interactions with the scaffold protein RACK1 [67, 68].

Similar to PDE4D5, PDE4D7 has been found to locate to the plasma membrane in a prostate cancer cell line and has been linked to regulation of cell proliferation [69]. Furthermore, in different cell types, i.e., human arterial endothelial cells, PDE4D7 was found to specifically regulate transcriptional responses to extracellular cues [70]. Based on these studies, neuronal PDE4D7 may also locate near the membrane to regulate cAMP-mediated relaying of extracellular signaling to an adaptive transcriptional response. Intriguingly, several single-nucleotide polymorphisms (SNPs) in the PDE4D7 and PDE4D5 promoter regions of the *PDE4D* gene showed significant associations with cognitive performance in humans [71]. Based on our data, it could be suggested that these SNPs, by influencing PDE4D isoform expression, may cause changes in neuronal plasticity (e.g., growing neurites) and thereby cause differences in cognition.

Regarding PDE4D9, a similar intracellular distribution as PDE4D3, i.e., perinuclear and under the plasma membrane, has been reported [72]. In the sub-membrane compartment, PDE4D9 can bind to and regulate the function of β_2_-adrenergic receptors [73]. As these receptors regulate neurite outgrowth and are also expressed in HT22 cells, PDE4D9 knockdown may contribute to a stronger or longer sustained signaling downstream of these receptors to facilitate neurite outgrowth [74, 75]. Functionally, PDE4D9 was also found to be phosphorylated by a multitude of kinases to regulate cAMP levels during mitosis [72] and could, therefore, halt cell cycle progression and induce neurite outgrowth upon knockdown. Moreover, PDE4D9 transcription is repressed by the scaffolding protein disrupted in schizophrenia 1 (DISC1), suggesting that DISC1 loss-of-function can induce increased PDE4D9 expression [76]. At the protein level, DISC1 directly binds and inhibits PDE4B and PDE4D, providing an additional manner to regulate PDE4D activity [77]. Interestingly, DISC1 loss-of-function mutations have been associated with psychiatric disorders associated with altered neuronal morphology [78]. These morphological changes may be partly explained by aberrant cAMP signaling caused by the impaired PDE4D9 transcription regulation and deficient PDE4(D) scaffolding by DISC1.

**Fig. 7:**
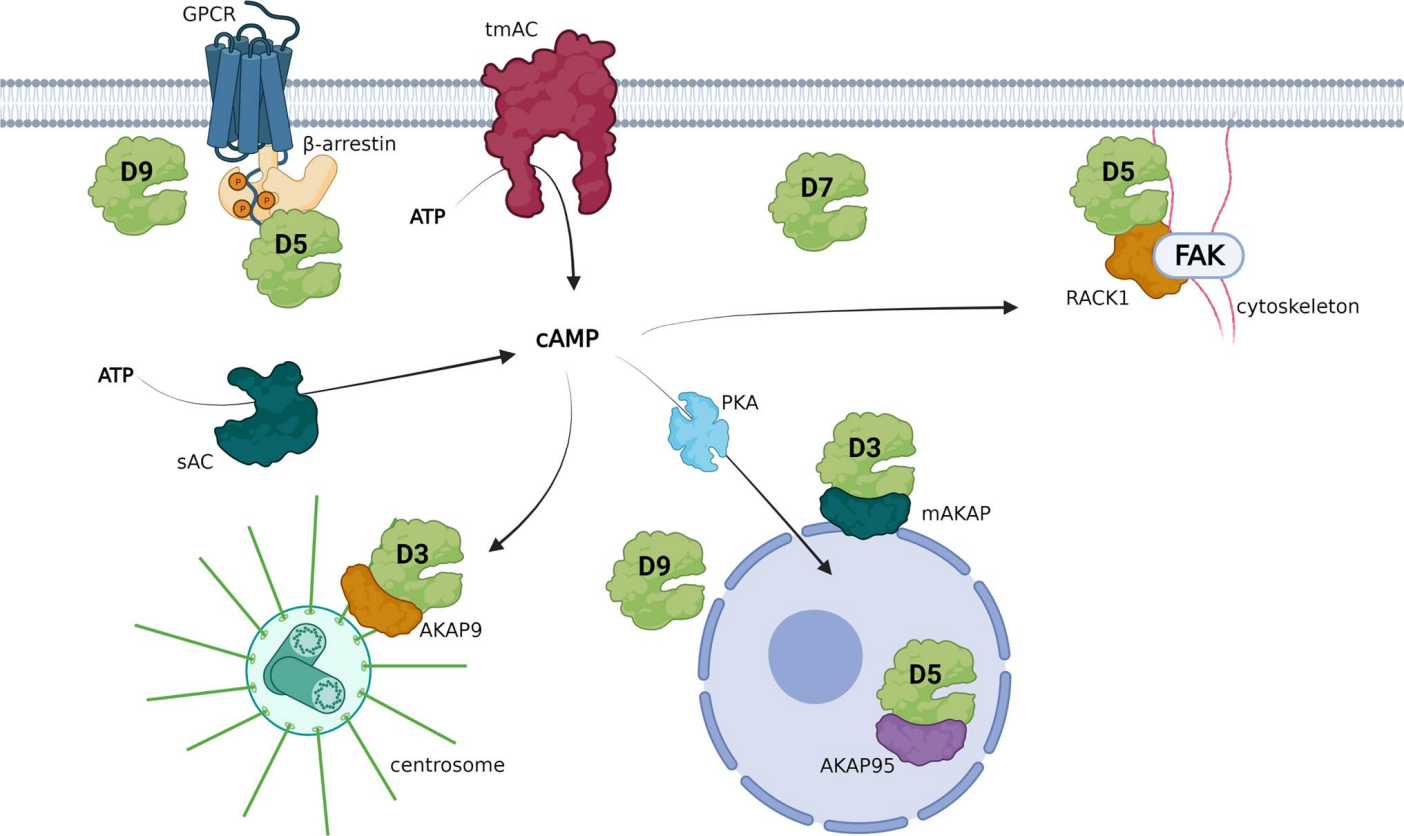
Intracellular positioning of specific PDE4D isoforms allows control of cAMP signaling involved in neuronal plasticity. PDE4D isoforms can regulate cAMP signaling at the site of synthesis (PDE4D9 near β-adrenergic receptors, PDE4D5 binding GPCR-recruited β-arrestin, and sub-membranous PDE4D7). Upon synthesis by transmembrane (tmAC) or soluble (sAC) adenylyl cyclases, cAMP signals can be relayed to centrosomal areas where PDE4D3 is sequestered by AKAP9 or to cytoskeletal areas where PDE4D5 is complexed to FAK by RACK1. This localization allows for PDE4D-mediated control of cell cycle progression and morphology changes. Moreover, cAMP-PKA-CREB signaling can be influenced by PDE4D isoforms in peri- and intra-nuclear locations (i.e., PDE4D3, PDE4D5, and PDE4D9) to control cAMP-mediated transcriptional responses. This image was created using Biorender

Thus, the long isoforms PDE4D3, -D5, -D7, and -D9 are well positioned in several cellular compartments to regulate (downstream) GPCR signaling, cAMP-PKA-CREB-mediated transcription, proliferation, cytoskeletal modulation, and local PKA-mediated phosphorylation events (Fig. 7). By genetic knockdown of separate PDE4D isoforms, local cAMP signaling is enhanced, which translates into a more complex morphology. Moreover, as PDE4D isoform knockdown also provided resilience against Aβ_1-42_-induced neurotoxicity, additional cellular mechanisms may be stimulated. The current findings indicate that genetic knockdown of a single PDE4D isoform is sufficient to induce these effects. This may imply that PDE4D isoform knockdown is not directly compensated for by other PDE4D isoforms, at least in the acute setting used here. Based on this rationale, follow-up studies would have to investigate potential synergistic actions of knockdown of multiple PDE4D isoforms. Previous studies have shown that inhibition of different PDE families, which would localize to different intracellular locations, can have synergistic effects [78, 79]. Possible additive effects of knockdown of multiple PDE4D isoforms would have to be revealed in such studies. However, pharmacological PDE4D inhibition, which would target multiple PDE4D isoforms, showed effects of similar potency as those observed for single isoform knockdown, making additive effects unlikely. Future studies should determine which (combinations of these) PDE4D isoforms, bound to which interactor proteins, regulate which local signaling module in the context of neuronal plasticity processes like neurite growth. Previously, the use of dominant-negative approaches has proven to be successful in elucidating the localization and function of specific PDE4 isoforms, and may therefore provide a useful tool for follow-up research [80, 81].

In general, long PDE4(D) isoforms share the ability to be phosphorylated by PKA which subsequently induces conformational changes leading to activation of these PDE4D isoforms [82, 83]. By adopting different conformational states, PDE4 enzymes can also exhibit different affinities toward inhibitors [13]. Historically, a high-affinity rolipram-binding state (HARBS) and low-affinity rolipram-binding state (LARBS) have been described, which can be bound specifically by different PDE4 types of PDE4 inhibitors [84, 85]. Long PDE4(D) can dimerize and thereby stabilize the HARBS conformation [86]. Interestingly, as inhibition of HARBS was found to stimulate neurite outgrowth while inhibition of LARBS did not [87], these functional effects may also be contributing to inhibition of long PDE4D isoforms. Additional support for the role of long PDE4D isoforms in regulating neuronal plasticity and protection against Aβ_1-42_-induced neurotoxicity was found by in vivo preclinical studies using shRNA-mediated knockdown of long PDE4D isoforms [18, 88, 89]. Reassuringly, these studies found dendritic complexity, CREB signaling, and cognitive performance to be enhanced upon PDE4D long-form knockdown. These effects were ascribed to knockdown of PDE4D4 and PDE4D5 as qPCR indicated decreased expression of these forms. However, expression effects on other long PDE4D isoforms (i.e., PDE4D7, -D8 and -D9) were not reported although the shRNA sequence used would be expected to also cause degradation of these other isoforms [88, 89]. Thus, knockdown of PDE4D7 and/or PDE4D9 might have contributed to the enhanced morphology complexity, CREB signaling and cognitive performance. The seemingly predominant role of long PDE4D isoforms in regulating neuroplasticity and cognition is further underlined by the association of mutations in *PDE4D* exons specific to long isoforms and the rare genetic disorder acrodysostosis, which is characterized by intellectual disability [90]. Strikingly, many of these mutations occur in protein domains that are unique to long PDE4D isoforms [91].

While the results presented here show robustly that silencing of long PDE4D isoforms specifically enhances neuronal plasticity, the question remains whether these specific long PDE4D isoforms also mediate processes contributing to the adverse side effects associated with PDE4D inhibition. The molecular mechanisms underlying these side effects remain to be defined but seem to be induced by PDE4(D) inhibition in both the emetic brainstem regions as well as the gastrointestinal system [13, 24, 92]. Emesis-inducing cAMP signaling in the area postrema in the brainstem may rely on signaling downstream of the glucagon-like peptide 1 receptor (GLP1R) [93, 94]. Thus, it can be argued that the PDE4D isoforms that mediate cAMP signaling induced by GLP1Rs should not be inhibited to prevent these emetic brainstem regions from being activated. Remarkably, PDE4D5 has been linked to GLP1 release, but a possible link between PDE4D and GLP1-induced signal transduction upon GLP1R activation remains to be elucidated [81]. Regarding peripherally regulated side effects, it has recently been described that non-selective PDE4 inhibition induces gastroparesis (i.e., delayed gastric transit), which could subsequently trigger nausea and emesis [92]. However, it remains to be elucidated whether long isoforms of the different PDE4 subtypes predominantly mediate these processes.

## Conclusion

In summary, this study shows that inhibition of individual long PDE4D isoforms specifically enhances neuronal plasticity, and that inhibition of these isoforms can yield resilience against Aβ-induced pathology in neurons. Hence, this target specification provides insights for the development of efficacious PDE4D inhibitors and supports the potential of PDE4D as a pharmacological target for the treatment of memory deficits in AD.

### Supplementary Information

Below is the link to the electronic supplementary material.

Supplementary file1 (XLSX 117 KB)

Supplementary file2 (DOCX 1805 KB)

## Data Availability

The datasets supporting the conclusions of this article are included within the article and its supplementary files.
