# Supplementary material for: Ablation of specific long PDE4D isoforms increases neurite elongation and conveys protection against amyloid-β pathology
Source: Cell Mol Life Sci. 2023 Jun 12;80(7):178. doi: 10.1007/s00018-023-04804-w (PMC10261250; doi:10.1007/s00018-023-04804-w)
Supplement: Supplementary file 2 — Supplementary file2 (DOCX 1805 KB) [file 18_2023_4804_MOESM2_ESM.docx]

# **Cellular and Molecular Life Sciences**

# **Supplementary Material**

# **Ablation of specific long PDE4D isoforms increases neurite elongation and conveys protection against amyloid-β pathology**

Dean Paes^1,2^, Melissa Schepers^1,2^, Emily Willems^1,2^, Ben Rombaut^1,2^, Assia Tiane^1,2^, Yevgeniya Solomina^1^, Amy Tibbo^3^, Connor Blair^3^, Elka Kyurkchieva^3^, George S. Baillie^3^, Roberta Ricciarelli^4,5^, Chiara Brullo^6^, Ernesto Fedele^5,7^, Olga Bruno^6^, Daniel van den Hove^1,8*^, Tim Vanmierlo^1,2*^, Jos Prickaerts^1*^

^1^Department of Psychiatry & Neuropsychology, School for Mental Health and Neuroscience, Maastricht University, Maastricht, the Netherlands.

^2^Department of Neuroscience, Biomedical Research Institute, Hasselt University, Diepenbeek, Belgium.

^3^Institute of Cardiovascular and Medical Sciences, College of Medical, Veterinary and Life Sciences, University of Glasgow, Glasgow, United Kingdom.

^4^Department of Experimental Medicine, Section of General Pathology, School of Medical and Pharmaceutical Sciences, University of Genoa, Genoa, Italy.

^5^IRCCS Opsedale Policlinico San Martino, Genoa, Italy.

^6^Department of Pharmacy, Section of Medicinal Chemistry, School of Medical and Pharmaceutical Sciences, University of Genoa, Genoa, Italy.

^7^Department of Pharmacy, Section of Pharmacology and Toxicology, School of Medical and Pharmaceutical Sciences, University of Genoa, Genoa, Italy.

^8^Department of Psychiatry, Psychosomatics and Psychotherapy, University of Würzburg, Würzburg, Germany

*Contributed equally

Corresponding author Jos Prickaerts: jos.prickaerts@maastrichtuniversity.nl

**Supplementary Table 1. Primer sequences and annealing temperatures used for qPCR.**

| **Transcript** | **Forward primer (5’-3’)** | **Reverse primer (5’-3’)** | **T_a_ (°C)** |
| --- | --- | --- | --- |
| Pde4d1 | GTCAAGCTGGAGCATCTCAGCC | TTCGTAAGCGCTTCACGGG | 63 |
| Pde4d3 | GCTCAAACCAGAGTGTTGGG | TTCGCAGCTCTTCCGTCATT | 58 |
| Pde4d4 | GAGCGCTACCTGTACTGCCG | ATGGGATCCAAGGGACTCCG | 58 |
| Pde4d5 | AATGGCTCAGCAGACGACAA | GGGGAGAGCTTGGGAGAAAC | 58 |
| Pde4d6 | AGCTGCTGAATTCCGTTCCA | GCCATTCAGGGTGTGGGAAT | 58 |
| Pde4d7 | CTCACCACCTGCCCTCAAAT | AGGCTCTCCTCACTCTCTCC | 58 |
| Pde4d8 | CCAGGACCATCTCCAAGAACTA | GCTGTCAGATCGGTACAGGAA | 58 |
| Pde4d9 | GTTCCCTGAGGACAACGGAG | TGCTTGGAGAATCAGCCCAG | 58 |
| 18s | ACGGACCAGAGCGAAAGCAT | TGTCAATCCTGTCCGTGTCC | 55 |
| Ppia | GCGTCTCCTTCGAGCTGTT | AAGTCACCACCCTGGCA | 55 |
| Ywhaz | GCAACGATGTACTGTCTCTTTTGG | GTACACAATTCCTTTCTTGTCATC | 49 |

**Supplementary Table 2. Oligonucleotide sequences annealed and ligated in the PX458 vector as gRNA against PDE4D isoforms.**

| **Isoform** | **Forward gRNA (5’-3’)** | **Reverse gRNA (5’-3’)** | **Frameshift frequency** (based on inDelphi algorithm) |
| --- | --- | --- | --- |
| Pde4d1 | CACCGCATCCGAGCATGGCGGGGTA | AAACTACCCCGCCATGCTCGGATG | 67,7% |
| Pde4d3 | CACCGTACATGCAACATAGGAGACG | AAACCGTCTCCTATGTTGCATGTAC | 89,4% |
| Pde4d4 | CACCGCCCGGGCGGTCAGCGAAGA | AAACTCTTCGCTGACCGCCCGGGC | 61,6% |
| Pde4d5 | CACCGAAGTGGATAATCCGCATGT | AAACACATGCGGATTATCCACTTC | 61,5% |
| Pde4d6 | CACCGTATTTATTGTCAGTGTCTTG | AAACCAAGACACTGACAATAAATA | 80,5% |
| Pde4d7 | CACCGATCTCGTACGGCGACTTTCT | AAACAGAAAGTCGCCGTACGAGAT | 85.9% |
| Pde4d8 | CACCGAGAACTAGAACAAGATTGCG | AAACCGCAATCTTGTTCTAGTTCTC | 73,7% |
| Pde4d9 | CACCGGTCTACAAGTTCCCTGAGG | AAACCCTCAGGGAACTTGTAGACC | 57,5% |

**Supplementary Table 3. Primers for HT22 genomic amplicon generation for the in vitro cell-free cleavage assay.**

| **Isoform** | **Forward primer (5’-3’)** | **Reverse primer (5’-3’)** | **Amplicon size (bp)** | **Fragment sizes (bp)** |
| --- | --- | --- | --- | --- |
| Pde4d1 | AGCTGATTCATTCGCTTCGC | AGGTCACAGGGATCGGTGAT | 681 | 283 + 398 |
| Pde4d3 | TCACCAGGACAATACTCGCC | CTTCTATGGAAATGCAGGCCA | 739 | 286 + 453 |
| Pde4d6 | TGCTGAATTCCGTTCCATTTTGG | AGCTTTAAAGACGAAGGTGGGAA | 720 | 261 + 459 |
| Pde4d9 | GAACTCCCTTCGGAAGAGCC | ATGCTCGTGTTTAGCCTCGT | 753 | 239 + 514 |

**Supplementary Table 4. sgRNA 58bp forward templates including T7 promoter sequence for the in vitro cell-free cleavage assay. sgRNA sequence in bold.**

| **Isoform** | **Forward sequence (5’-3’)** |
| --- | --- |
| Pde4d1 | CCTCTAATACGACTCACTATAGG**CATCCGAGCATGGCGGGGTA**GTTTAAGAGCTATGC |
| Pde4d3 | CCTCTAATACGACTCACTATAGG**TACATGCAACATAGGAGACG**GTTTAAGAGCTATGC |
| Pde4d6 | CCTCTAATACGACTCACTATAGG**TATTTATTGTCAGTGTCTTG**GTTTAAGAGCTATGC |
| Pde4d9 | CCTCTAATACGACTCACTATAGG**CCTCAGGGAACTTGTAGACC**GTTTAAGAGCTATGC |


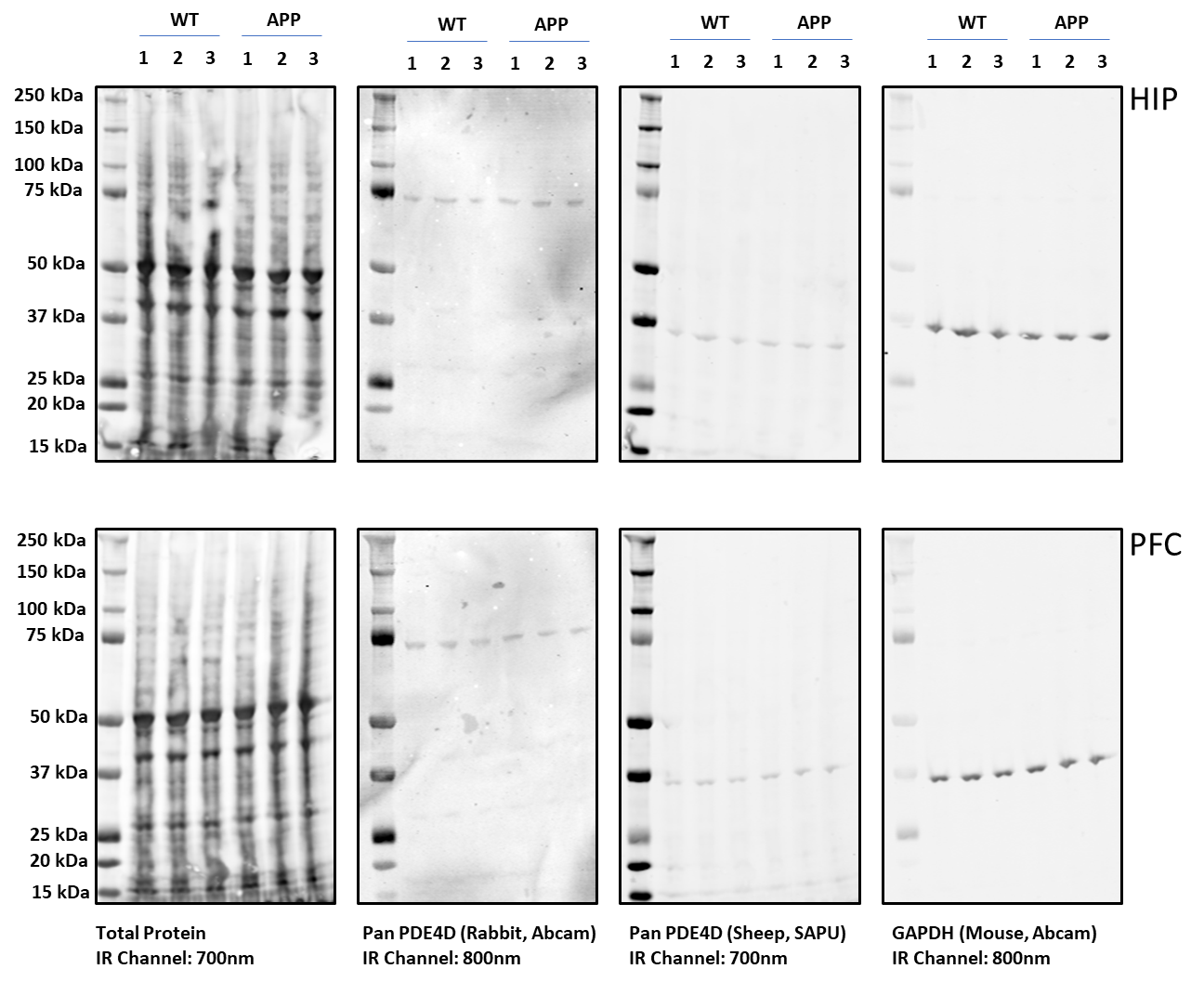
**Supplementary Figure 1. Western blot scans used for quantification as depicted in Figure 2A.**


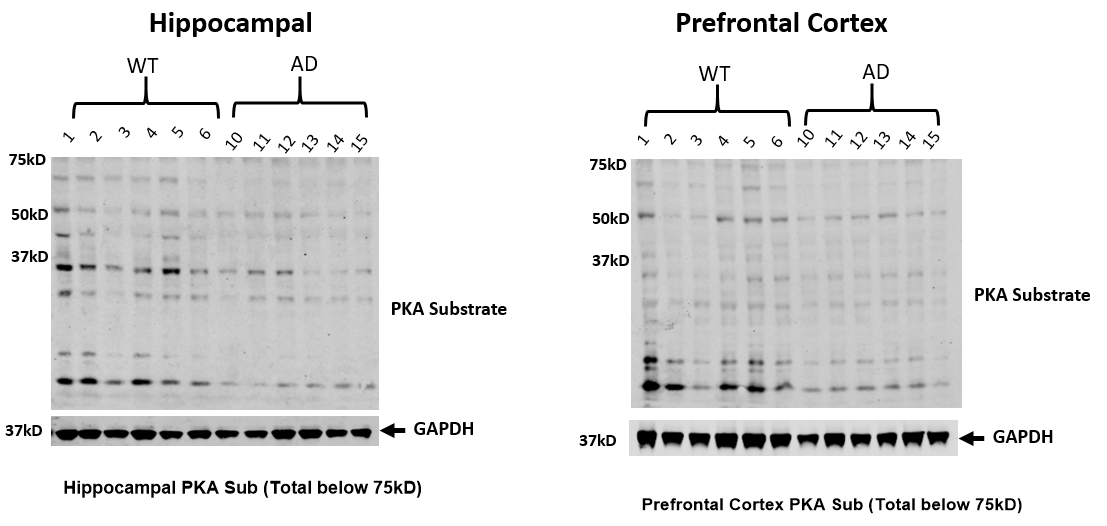


**Supplementary Figure 2. Western blot scans used for quantification as depicted in Figure 2C.**


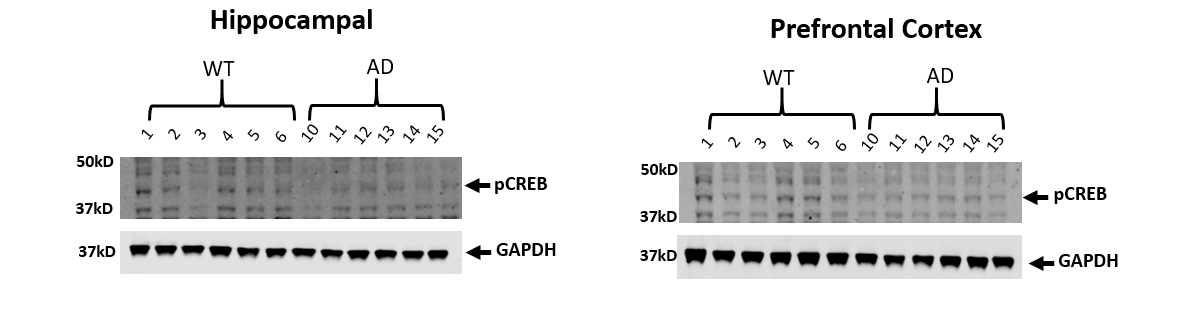


**Supplementary Figure 3. Western blot scans used for quantification as depicted in Figure 2D.**


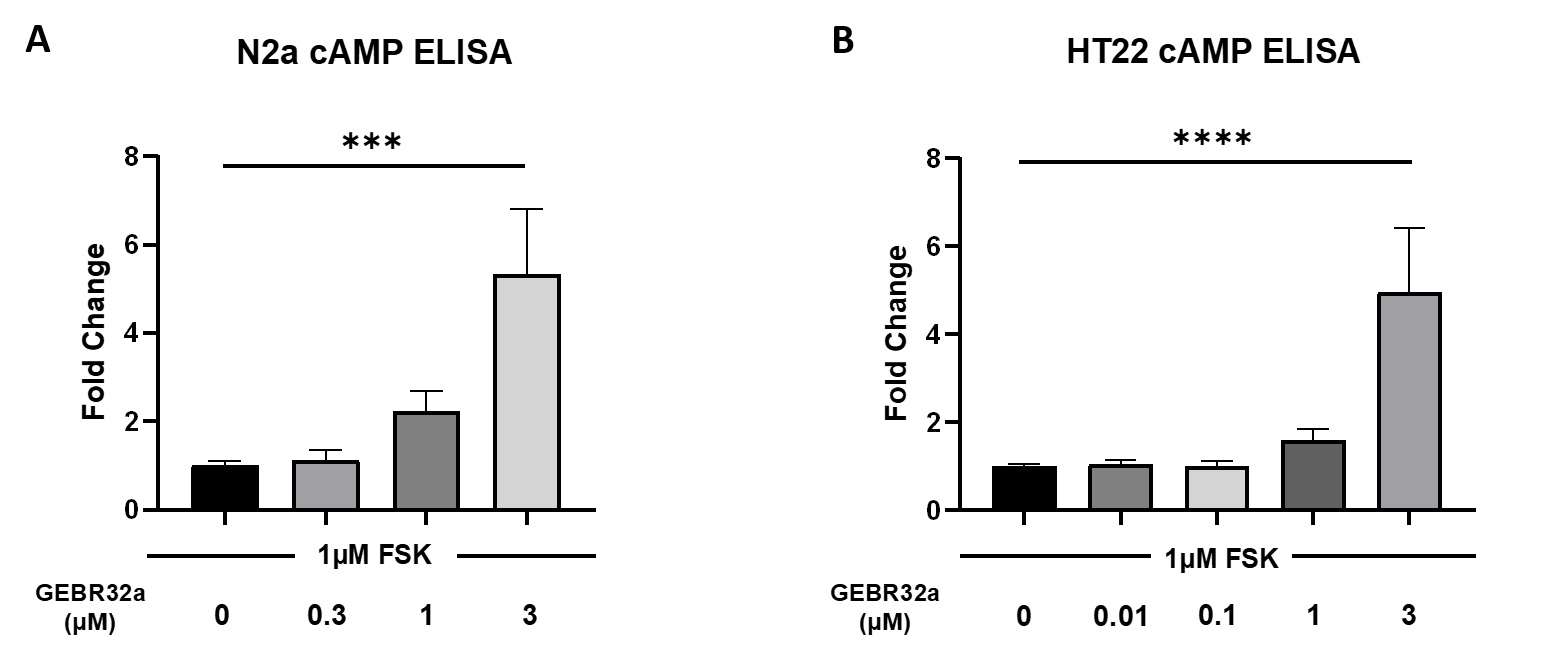


**Supplementary Figure 4. Determination of intracellular cAMP concentration upon forskolin (FSK) stimulation to increase baseline cAMP levels, and concomitant GEBR32a treatment in N2a (A) and HT22 (B) cells** (Dunnett’s post-hoc: ***P<0.001, ****P<0.0001; n≥4/condition). Data is presented as mean + SEM.


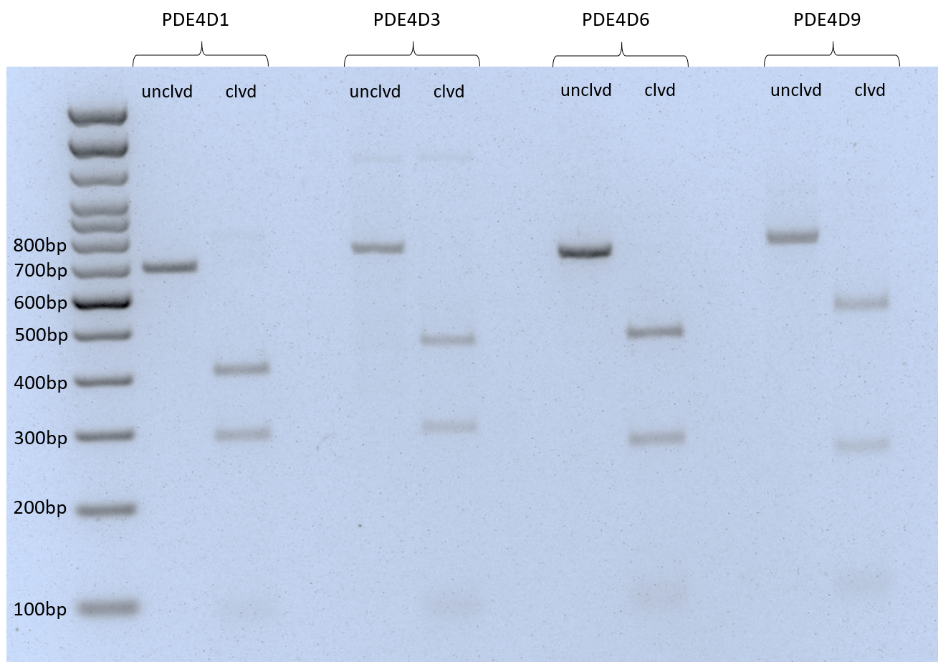


**Supplementary Figure 5. A cell-free in vitro cleavage assay indicates that the sgRNA designed for PDE4D1, PDE4D3, PDE4D6, and PDE4D9 induces a double stranded break at the predicted site.** sgRNAs for PDE4D1 and PDE4D6 were evaluated in this assay as these target (super)short PDE4D isoforms that did not show a biological effect in the neurite outgrowth assay. sgRNAs for PDE4D3 and PDE4D9 were evaluated as knocking down these long isoforms showed the largest effect in the neurite outgrowth assay. For each sgRNA, the figure shows an uncleaved (unclvd) fragment in lane 1, and two cleaved (clvd) fragments in lane 2. Fragment size is based on primer design as shown in supplementary table 3. The sgRNA itself appears slightly near the 130bp position.
